# COVID-19 in Light of Seasonal Respiratory Infections

**DOI:** 10.3390/biology9090240

**Published:** 2020-08-20

**Authors:** Irina Kiseleva, Elena Grigorieva, Natalie Larionova, Mohammad Al Farroukh, Larisa Rudenko

**Affiliations:** Federal State Budgetary Scientific Institution “Institute of Experimental Medicine”, 197376 Saint Petersburg, Russia; epgrigorieva@gmail.com (E.G.); nvlarionova@mail.ru (N.L.); mouhammad1farroukh@gmail.com (M.A.F.); vaccine@mail.ru (L.R.)

**Keywords:** viral respiratory infections, pandemics, epidemics, influenza, COVID-19

## Abstract

A wide diversity of zoonotic viruses that are capable of overcoming host range barriers facilitate the emergence of new potentially pandemic viruses in the human population. When faced with a new virus that is rapidly emerging in the human population, we have a limited knowledge base to work with. The pandemic invasion of the new SARS-CoV-2 virus in 2019 provided a unique possibility to quickly learn more about the pathogenesis of respiratory viruses. In this review, the impact of pandemics on the circulation of seasonal respiratory viruses is considered. The emergence of novel respiratory viruses has often been accompanied by the disappearance of existing circulating strains. Some issues arising from the spread of pandemic viruses and underlying the choices of a strategy to fight the coronavirus infection are discussed.

## 1. Introduction

With a wide diversity of zoonotic viruses capable of overcoming host range restrictions and acquiring the ability to spread in an immunologically naive population, the risk of the emergence of a new potentially pandemic virus in the human population is always present [1].

For many years, the term “pandemic” has been associated with influenza. A claim of the pandemic potential of an influenza virus rests on three premises. For a strain of influenza virus to cause an epidemic or pandemic, it should be (i) antigenically novel to the human immune system; (ii) virulent to the human host; (iii) person-to-person transmissible [2]. Taken separately, each of these prerequisites is necessary, but not sufficient, for the virus to become a pandemic strain. Only the combination of all three qualities provides the influenza virus with pandemic potential.

All of this fully applies to any pandemic virus of a different origin. As the tragic history of recent months has shown, another virus can also act as a pandemic virus that is not less, but rather significantly more dangerous than influenza viruses.

At the end of December 2019, China reported a cluster of pneumonia cases of unknown causes in Wuhan, Hubei province. Soon after, Chinese scientists identified a novel beta-coronavirus as the most likely causative agent. The virus is now referred to as severe acute respiratory syndrome coronavirus 2 (SARS-CoV-2), and the disease is called COVID-19 (coronavirus disease 2019) [3]. SARS-CoV-2 properties satisfy the three basic requirements that enable a strain to become a pandemic: it is new to the human immune system, highly virulent to infected persons, and highly transmissible—less transmissible than measles but more contagious than influenza.

Globally, since 31 December 2019 and as of 12 August 2020, there have been 20,162,474 confirmed cases of COVID-19, including 737,417 deaths, reported to the World Health Organization (WHO) [4]. The country with the highest mortality (14.9%) is the United Kingdom. The mortality rate in the majority of countries worldwide is much lower.

## 2. Impact of Influenza Pandemics on the Circulation of Seasonal Respiratory Viruses

Wars, stresses, and prolonged force majeure situations upset the delicate balance in host-pathogen interactions. Nevertheless, not only wars but also global outbreaks are stressful for society. What happens to other infectious diseases during global disasters such as pandemics?

There is a wide range of respiratory pathogens associated with human infections. To better understand the threat that these viruses pose to humankind, it is often valuable to study newly identified viruses, not in isolation, but in comparison with other related viruses [1]. In this section, we will briefly discuss the relationships between newly emerged pandemic viruses and circulating seasonal strains.

The first virologically documented pandemic, the Spanish flu, occurred in 1918–1919 [5]. It was caused by the influenza A (H1N1) virus. According to various estimates, this pandemic, unprecedented in its severity, killed between 40 and 50 million people and caused a huge number of severe forms of the disease. Thirty percent of the world’s population became sick. In subsequent years, the Spanish influenza A (H1N1) moved into the category of seasonal pathogens. The virus acquired mutations that allowed it to escape the population’s immunity and cause influenza epidemics annually.

Unfortunately, we were unable to find accessible literature on the prevalence of other respiratory viral infections during the most destructive pandemic of all, the 1918 influenza pandemic. No documentary evidence regarding the pro et contra spread of other respiratory illnesses could be found. However, a huge range of publications describing the situation with the spread of common respiratory infections during the following pandemics, in 1957, 1968, and 2009, are available.

In 1957, a new pandemic began, caused by the Asian influenza A (H2N2) virus [6,7], killing 1.5 million people. Asian influenza viruses circulated for 11 years, causing annual epidemics.

A new influenza A (H3N2) pandemic virus, called the Hong Kong virus, replaced the Asian influenza virus in 1968 [6,8]. It killed 1 million people. Drift variants of the influenza A/Hong Kong (H3N2) virus continue to circulate to date.

In 1977, the subtype influenza A (H1N1) virus again caused morbidity among young, immunologically naive people [8]. Studies have shown that this virus was not genetically different from the A (H1N1) virus circulating in the 1950s.

It is noteworthy that the emergence of each new pandemic virus was regularly marked by the disappearance of its pandemic predecessor, drifting in seasonal epidemics. There is an explanation of the mysterious phenomenon of the displacement of a previously circulating virus by a new pandemic virus, according to which the mechanism leading to the elimination of the previous pathogen is an increase in the production of human antibodies to the conserved stem of viral hemagglutinin protein. These antibodies are enough to displace an obsolete virus [9,10].

An exception is the joint circulation of viruses A (H3N2) and A (H1N1) after 1977 when virus A (H1N1) returned to the community, and was unable to displace virus A (H3N2). Both subtypes alternately or simultaneously circulated among people until 2009.

In 2009, the serotype A (H1N1) viruses that drifted since 1977 were supplanted by a completely new virus of the same serotype A (H1N1)pdm09, which caused a new moderate pandemic [11,12]. Virus A (H1N1)pdm09 has moved into the category of seasonal viruses and continues to circulate with viruses A (H3N2) to date.

The evolution of influenza viruses is related to all of the above. What happens to seasonal respiratory viruses in the interpandemic and pandemic periods? As an example, we consider the situation covering the period before and after the last influenza pandemic of 2009 (2007–2012). In sum, influenza A (H1N1)pdm09 pandemic viruses had distinct effects on the circulation of other respiratory viruses when they first appeared. On the contrary, no effect was seen when novel A (H1N1)pdm09 strains evolved from pandemic to seasonal viruses. When pandemic influenza virus strains just replaced the circulating seasonal influenza virus strains, seasonal influenza A (H3N2) (0.3%) and B (2.0%) infections were hardly detected. However, the circulation of other respiratory pathogens remained unchanged [13].

Globally, seasonal influenza has demonstrated wide variations in incidence depending on the epidemic season and the antigenic properties of the circulating strains. In contrast, the yearly incidence rates for such noninfluenza pathogens, such as respiratory syncytial virus, are fairly stable. At the same time, influenza virus detection had always been significantly below that of the respiratory syncytial virus in influenza seasons before 2009 [14]. The 2009 pandemic significantly altered the detection rates of respiratory viruses, establishing influenza viruses as the major respiratory pathogens worldwide [14]. As an example, a study of the circulation of respiratory viruses other than influenza during the 2009 pandemic in Spain revealed that apart from influenza A (H1N1)pdm09 viruses, other respiratory viruses were detected in 39.9% of patients. Co-infection of two or more respiratory viruses was observed in approximately 15% of cases. The highest detection rates were obtained for human rhinovirus and respiratory syncytial virus, representing 43.7% and 31.4% of positive noninfluenza samples [14].

It has been hypothesized that at least in some European countries, a viral interference of human rhinovirus could have delayed the epidemic evolution of the pandemic influenza A (H1N1)pdm09 virus in the fall of 2009. Similarly, the influenza A (H1N1)pdm09 virus could interfere with another respiratory pathogen, a respiratory syncytial virus, in the same period [14].

Some authors have postulated that a possible interference of other respiratory viruses such as human rhinovirus and respiratory syncytial virus with influenza A (H1N1)pdm09 virus during the pandemic could change the epidemic wave of pandemic influenza [15,16,17].

## 3. The Role of Mixed Infections in Etiology of Pulmonary Diseases

**Figure 1:**
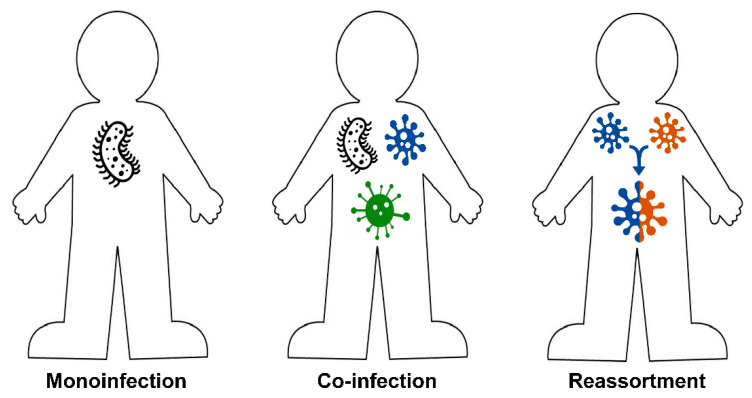
Host-pathogen interactions.

Mono-infections are not the only infections that can be found in nature. The co-infection of more than one pathogen in one host is common across viruses and occurs more commonly than would be expected by chance in nature [18,19]. Different variations of interactions of circulating pathogens exist in nature. It could be a (i) single infection (one pathogen, one host) [14,20]; (ii) mixed infection (co-infection), when two or more antigenically distinct pathogens infect one host [21,22,23,24,25,26]; (iii) reassortment of two or more viruses in one host, when an individual is co-infected with two or more of the same type of virus, for example, influenza A viruses, resulting in reassortant progeny [27,28,29] (Figure 1). The second variant of host-pathogen interactions—co-infection—is most closely related to the focus of this paper.

The mixed etiology of pulmonary infections is frequent, with co-infections of pathogenic respiratory influenza-like viruses and bacteria in respiratory secretions. The clinical significance of these viral-bacterial co-infections has been a controversial topic for a long time [30]. Viruses can alter the host’s susceptibility to bacterial infections both by altering the environmental conditions in the lung to favor bacterial replication and by suppressing the host’s defense mechanisms to prevent the clearance of the bacteria [31]. Understanding the synergy or antagonism between viruses and bacteria will facilitate the design of novel therapeutic approaches to prevent the mortality associated with bacterial co-infections [32].

The human respiratory tract hosts a diverse community of cocirculating viruses that are responsible for acute respiratory infections. In the respiratory airways, the ecological niche contains viruses and bacteria that periodically circulate and enter interspecific interactions [33,34,35]. An example of the interaction between viruses and bacteria is the relationship between influenza virus and pneumococcus, leading to about a 100-fold increase in susceptibility to pneumococcal pneumonia after influenza infection [36]. This shared niche also facilitates virus-virus interactions [33]. During a seasonal rise in the incidence of influenza, a few antigenically distinct viruses circulate, the ratio of which varies annually. Seasonal influenza consists of variable mixes of influenza A (H3N2), A (H1N1)pdm09, and the two B virus lineages.

Evidence of virus-virus interactions between 11 human respiratory viruses has recently been presented [33]. The experimental models of respiratory virus co-infections have demonstrated several interaction-induced effects, from an enhanced or reduced viral growth to the attenuation of disease. In the case of recirculation, seasonal influenza-like viruses, and a ubiquitous common-cold-like virus, a short-lived protective effect, such as that induced by interferon, was demonstrated [37,38]. It has also been shown that cell fusion induced by certain viruses may enhance the replication of others in co-infections. However, despite the epidemiological, clinical, and experimental indications of interactions among respiratory viruses, quantitatively robust evidence is still lacking [33].

The cocirculation of different respiratory viruses can lead not only to cooperative but also to competitive forms of virus-virus interactions. It is believed that such interactions occur, for instance, among influenza and rhinoviruses [33]. It can also be assumed that competitive viruses are built between viruses, where an epidemically stronger rival wins. In such cases, the circulation of one viral pathogen may reduce the likelihood of or delay infection by another. Thus, the pandemic A (H1N1)pdm09 influenza virus delayed infection with respiratory syncytial virus. The 2009 respiratory syncytial virus infections were first noticed only when the infection with the pandemic swine influenza began to decline. Interestingly, in 2010 to 2011, infections with respiratory syncytial virus were observed together with the pandemic swine influenza infection and may reflect the conversion of the pandemic influenza virus from a pandemic virus to a regular seasonal influenza virus [39]. In other cases, such interactions can have large consequences, aggravating the course of diseases. Therefore, there is a clinical need for a robust investigation into co-infection in patients with COVID-19.

As noted above, pandemic influenza viruses displace seasonal influenza strains; however, the circulation of other respiratory pathogens such as rhinovirus and the respiratory syncytial virus remains at the interpandemic level. What happens to these seasonal respiratory viruses in the coronavirus pandemic?

## 4. Impact of the COVID-19 Pandemic on the Circulation of Seasonal Respiratory Viruses

**Figure 2:**
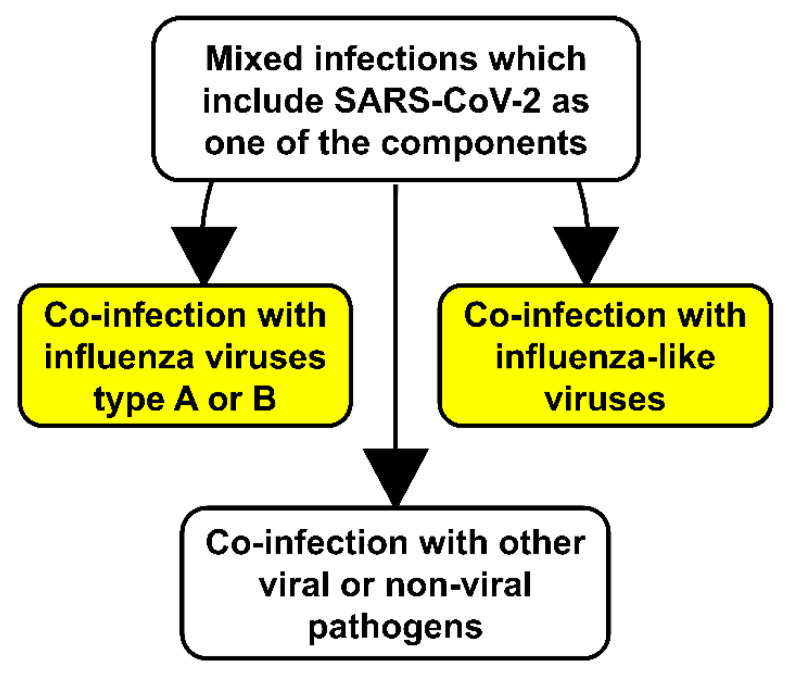
The nature of mixed infections of SARS-CoV-2 with other pathogens in the COVID-19 pandemic.

Humans are constantly being inoculated with various microorganisms resident in the upper respiratory tract and by inhaled aerosols [31]. Mixed infections significantly contribute to the insufficient control of respiratory infections. A single infection may be caused by the simultaneous synergistic interaction of a variety of pathogens. In the context of SARS-CoV-2, in the COVID-19 pandemic, individuals may be co-infected with (i) influenza virus; (ii) another respiratory virus; (iii) other pathogens such as nonrespiratory viruses, bacteria including *Mycobacterium tuberculosis*, fungi, and mycoplasma (Figure 2). Each of these variants may negatively influence COVID-19 treatments. An important question raised by many scientists is the following: should we expect an increasing or decreasing number of co-infections between SARS-CoV-2 and other respiratory pathogens, and what do we observe now?

COVID-19 can occur with other viral infections. Some of these co-infections can be effectively treated, while only supportive treatment is the mainstay of treatment for others [40].

Most publications describing co-occurring infections in which COVID-19 is involved focus on influenza and influenza-like diseases. There are only a few papers on other infections. In this review, we have strictly confined ourselves to influenza and other viral respiratory infections.

### 4.1. Co-Infection of SARS-CoV-2 with Influenza Viruses

According to the WHO, as of 22 June 2020, global influenza activity is at lower levels than expected for this time of the year. In the Northern Hemisphere, influenza activity returned to inter-seasonal levels, while in the Southern Hemisphere, the influenza season has not started yet [41]. Most countries in the Caribbean and Central America reported to the WHO sporadic influenza detections, though many areas are experiencing rises in COVID-19 cases [41]. The WHO warns that its information should be interpreted with caution because of the possible influences of both COVID-19 activity and distance measures. During the epidemic of COVID-19 in Beijing (China), influenza viruses, especially influenza type A viruses, accounted for a large proportion of respiratory virus infections [42].

A recent study revealed that Wuhan citizens with documented mixed infections of SARS-CoV-2 and influenza A or B did not appear to show a more severe clinical condition; they displayed similar clinical characteristics to those patients with SARS-CoV-2 infections only [43].

Some believe that SARS-CoV-2 and influenza virus co-infection is rare [23]. Globally, there has been a limited number of COVID-19-influenza co-infected patients reported from different countries around the world. For instance, a study performed in Istanbul, Turkey revealed that only 0.54% out of 1103 COVID-19 patients were diagnosed as co-infected with influenza A or B viruses [23]. Co-infections with influenza A virus were reported in the USA, Turkey, Germany, Iran, China, Japan, and Spain [21,23,40,43,44,45,46,47,48,49,50]; co-infections with influenza B virus were reported in Turkey, China, and Spain [23,43,44]; simultaneous influenza A and B and COVID-19 infections was detected in China and Spain [44,51].

In any situation, influenza preparedness measures are crucial, especially for high-risk groups, and the influenza vaccination coverage rate must be at least 70% [52]. COVID-19 measures may reduce the number of cases of influenza infections. Seasonal influenza cases in the Northern Hemisphere usually peak in February and tail off by the end of May. Unusually, this year, lab-confirmed cases of influenza dropped dramatically in early April, soon after the WHO declared the COVID-19 pandemic. It has been hypothesized that the influenza season was cut short and that influenza incidence was substantially reduced by control measures aimed at interrupting SARS-CoV-2 transmission [53].

In a WHO press conference on 15 June 2020, the WHO Director-General Dr. Tedros A. Ghebreyesus said, “Despite the ongoing global response to the COVID-19 pandemic, we cannot lose sight of other significant public health issues including influenza. Influenza affects every country every year and takes its deadly toll. As we enter the Southern Hemisphere influenza season and begin planning for the Northern Hemisphere season, we must ensure that influenza remains a top priority” [54]. Dr. Tedros A. Ghebreyesus noted that today we face a dramatic decrease in the number of specimens tested for influenza globally. These disruptions may have short- and long-term effects such as loss of capacities to detect and report new influenza viruses with pandemic potential.

Thus, SARS-CoV-2 may significantly affect infections with other respiratory viruses including influenza viruses. However, it is difficult to distinguish its direct impact on reducing respiratory infections because of the effect of declining influenza surveillance as a result of the COVID-19 pandemic.

### 4.2. Co-Infection of SARS-CoV-2 with Influenza-like Viruses

Besides influenza viruses, other respiratory viruses play a significant role in epidemiological processes. Recently, respiratory viruses showed different levels of mixed infections with SARS-CoV-2 [55]. In light of the foregoing information, the COVID-19 pandemic will probably develop according to one of two scenarios: (i) SARS-CoV-2 viruses will cocirculate with other viral pathogens, or (ii) SARS-CoV-2 viruses will displace other respiratory viruses.

The latest evidence suggests that unlike co-infection rates with influenza viruses, which demonstrated some reduction in activity, the co-infection rates between SARS-CoV-2 and other respiratory pathogens are higher than expected [56]. A study performed in California (USA) revealed that, of patients with confirmed SARS-CoV-2 infections, 20.7% were positive for one or more other pathogens; the most common were rhinovirus, enterovirus, respiratory syncytial virus, and seasonal coronavirus [57]. In a study performed in China, among 32 confirmed COVID-19 cases, 14 patients were infected with other pathogens, including viral, bacterial, and fungal infections. Four of them were infected with respiratory viruses-respiratory syncytial virus, human parainfluenza virus, human metapneumovirus, and rhinovirus [58].

During the beginning of the COVID-19 spread in Japan, influenza and human metapneumovirus were prevalent among children (the study was performed in the Furano region). Three children were diagnosed with COVID-19, two of whom were co-infected with influenza and human metapneumovirus [59].

Some believe that co-infection with two respiratory pathogens is uncommon in adults and mainly inherent in children [26]. Nonetheless, a mixed infection of metapneumovirus and SARS-CoV-2 was detected in an adult patient in Providence (RI, USA) [26].

In sum, among the concomitants of SARS-CoV-2 pathogens, respiratory syncytial virus and rhinovirus prevail. Respiratory syncytial virus and rhinovirus are the most frequent causes of acute respiratory infections, with especially severe diseases in young children and the elderly. Primary respiratory syncytial virus infections may lead to severe bronchiolitis and pneumonia [60,61]. Rhinovirus usually causes rhinopharyngitis but may also cause illness of the lower respiratory tract and asthma exacerbations and has been associated with severe acute lower respiratory tract infections [60,61]. The post-infection immunity to these infections is short-lasting, which leads to the repeated circulation of viruses in the same population. The co-infection of such seasonal pathogens, along with influenza viruses, with the new pandemic SARS-CoV-2 can aggravate the course of the disease and represents a challenge for its diagnosis and treatment [52,62,63].

### 4.3. Co-Infection of SARS-CoV-2 with Other Viral and Nonviral Pathogens

Current evidence does not support a high rate of bacterial co-infections in patients with COVID-19 [24]. On the contrary, a low proportion of COVID-19 patients have bacterial co-infections [55]. Bengoechea and Bamford [3] noted that the limited reference to nonviral co-infections in COVID-19 is surprising. We found that only a few publications described co-infections with nonviral pathogens. In particular, there is limited information available regarding the mixed infections of SARS-COV-2 with bacterial and fungal infectious agents, including *Mycoplasma pneumonia* [64,65], *Mycobacterium tuberculosis* [66,67,68], *Legionella pneumophila* [69], and *Aspergillus fumigatus* [70,71]. Nonrespiratory viral pathogens, such as cytomegalovirus [72] and HIV [73], also did not play a crucial role in viral-viral mixed infections. Therefore, there is a paucity of data to support the association of SARS-CoV-2 and these pathogens with the severity of co-infection.

## 5. On the Way to the Anti-COVID-19 Vaccine

It is strongly hoped that this effort in developing an anti-COVID-19 vaccine will be successful. Almost 150 teams all over the world are working hard with a diverse set of strategies and platforms to develop a potent and harmless vaccine against COVID-19. As of 10 August 2020, 139 vaccines are in preclinical evaluation, and 28 are in phase 1, 2, or 3 clinical trials [74].

However, some caution is prudent, and the gains that have been recently made in understanding the interactions between the host immune system and viruses should be fully exploited [75]. Most vaccines go through years of tests before they appear on the market. Some authors suggest that the first vaccines may not prevent COVID-19 infection because, generally, vaccines aim to protect people against disease, not necessarily the infection itself [76]. Such a vaccine will prevent people from getting sick or dying but will not stop them from catching the coronavirus. Taking into account the fact that the vaccine may not prevent infection with the SARS-CoV-2 virus, we have to be warned that some coronavirus vaccinations might harm more than help those who are desperate for protection. A coronavirus vaccination could lead to negative consequences, including enhancing the negative effect of the infection in a person instead of mitigating it [77]. The “cytokine storm,” an uncontrolled over-production of soluble markers of inflammation, appears to be one of the most dangerous and potentially life-threatening events related to COVID-19 [78]. Infection with the wild-type SARS-CoV-2 virus followed by vaccination may induce a severe disease [79].

Safety is a primary goal for any vaccine; potential risks associated with vaccine development for COVID-19 are described in several publications [77,79,80,81,82,83]. Some authors suggest that vaccinations could increase the severity of subsequent SARS-CoV-2 infections. This has happened with vaccines against feline coronavirus [77]. There are two syndromes described for vaccine-enhanced diseases, antibody-dependent enhancements (a process in which a virus leverages antibodies to aid infection), and the vaccine-associated enhanced respiratory disease (cell-based enhancement that includes allergic inflammation).

Immunological priming could involve autoimmunity in lung tissues due to previous exposure to the coronavirus spike protein. Immunogenic viral peptides that match human proteins are good candidates for pathogenic priming peptides by analogy with the immune system reaction known as an immune enhancement. The homology between human and viral proteins is an established factor in viral- or vaccine-induced autoimmunity [81,83].

Thus, researchers must prove whether or not their vaccines cause the same types of immune system malfunctions that have been observed in the past.

Unfortunately, no specific antiviral therapy for COVID-19 exists; no licensed vaccine is available yet. Therefore, scientists put forward a wide variety of theories regarding which of the available chemical and immunobiological preparations may be used in the fight against this disease.

Since the early stages of the COVID-19 pandemic, a possible role of vaccination with a live attenuated vaccine against tuberculosis (bacille Calmette-Guérin vaccine, BCG) in preventing novel coronavirus infections has been discussed in numerous publications [84,85,86,87]. Based on the hypothesis that BCG can enhance the reactivity of the innate immune system, some researchers have speculated that the BCG vaccine may be used as a preventive and/or therapeutic measure against COVID-19.

Nonetheless, the results were conflicting, and later studies did not confirm a correlation between COVID-19 occurrence and BCG vaccination rates. After a detailed analysis of all available data, it was concluded that there is no reasonable evidence to recommend BCG vaccination for the prevention of COVID-19 [88,89]. The official position of the WHO is that there is no evidence that the BCG vaccine protects people against infection from the COVID-19 virus [90]. However, some clinical trials aiming to determine whether a BCG vaccination could protect against COVID-19 infections are in progress [91,92].

We also must remember that influenza remains an ever-present cause of disease and death globally [93]. Patients co-infected with SARS-CoV-2 and influenza virus have a higher risk of poor outcomes. Given the potential for influenza-COVID-19 co-infections globally [23,94], especially during the influenza season, it has been suggested that influenza vaccinations could be used to indirectly control COVID-19 [50,52,95]. Of course, the influenza vaccine will not fully protect against coronavirus infection, but a vaccination with a proven and safe influenza vaccine, especially a live one, will affect the incidence of mixed influenza-COVID-19 infections and thus reduce the severity of coronavirus infections.

Some authors suggested that this fall, the influenza vaccine for larger groups of the population should be recommended to simplify clinicians’ work when the second wave of COVID-19 is expected in parallel with the start of the influenza season [23,96]. Other researchers are not optimistic [80,97,98]. In any case, understanding the relationship between COVID-19 and influenza is very important.

Salem and El-Hennawy [95] hypothesized that immunity against influenza infections may foster immunity against SARS-CoV-2. This suggestion is supported by studies showing a cross-reactivity of immunity between influenza virus and coronavirus due to the similarity in their structures. The influenza vaccination itself would also generate sustained immunity that may enhance immunity against SARS-CoV-2. The authors suggest that the immunity induced by the influenza virus has more beneficial effects for COVID-19 than those suggested for BCG or MMR (measles, mumps, and rubella) vaccines. More than 500 million people are vaccinated against the flu every year based on recommendations from the WHO on the composition of influenza virus vaccines [54,99]. According to [95], given the high safety of the influenza vaccine confirmed by decades of vaccination of millions of vaccinees, influenza vaccines can be used to minimize the severity of the COVID-19 disease.

## 6. Conclusions

The pandemic invasion of a new SARS-CoV-2 virus in 2019 provided a unique possibility to learn more about the pathogenesis of respiratory viruses. When faced with a new virus that is rapidly emerging in the human population, we have a limited knowledge base to work with. The clock is ticking, and we have to learn quickly about this new virus while simultaneously using our knowledge of previous influenza outbreaks that have much in common with the recent COVID-19 outbreak [93,100]. Ozawa and Kawaoka [101] have suggested that the “complete control of influenza viruses seems impossible.” It seems that it is not futile to think that complete influenza eradication is possible in the near future. As for today, the COVID-19 pandemic is far from over, and as with influenza, we are far from defeating it. Meanwhile, nobody can predict anything. We are just at the beginning of a long and winding road.
